# Biomechanics of PHILOS plates in Vancouver B1 periprosthetic femoral fracture

**DOI:** 10.3389/fbioe.2023.1282128

**Published:** 2023-11-17

**Authors:** Changjun Yun, Wenjie Qian, Jie Zhang, Wen Zhang, Jinpeng Lv

**Affiliations:** ^1^ Departmeut of Orthopaedics, The Affiliated Wujin Hospital of Jiangsu University, Changzhou, China; ^2^ The Wujin Clinical College of Xuzhou Medical University, Changzhou, China; ^3^ Orthopedic Institute, Soochow University, Suzhou, China; ^4^ School of Pharmacy, Changzhou University, Changzhou, China

**Keywords:** femur, periprosthetic fracture, PHILOS plate, internal fixation, finite element analysis, biomechanics

## Abstract

**Objective:** To investigate the clinical efficacy of PHILOS plates in the treatment of Vancouver B1 periprosthetic femoral fracture (PFF) and to validate its biomechanical reliability via finite element analysis and mechanical testing on the Synbone femoral models.

**Methods:** Ten males and eight females with Vancouver B1 PFF who underwent PHILOS plate fixation between September 2017 and January 2022 were selected. The average age was 72.61 ± 8.19 years, with a range of 57–86 years old. X-ray films were taken to assess the fracture healing situation around the femoral prosthesis as well as the position of the PHILOS plates and femoral prosthesis. Two different plates (the PHILOS plate and the Cable GTR plate) were used for fixation, and the differences in biomechanical stability of the two fixation methods were compared using finite element analysis and mechanical testing on the Synbone femoral models to validate the biomechanical dependability of the PHILOS plate.

**Results:** All 18 cases were followed for at least 1 year, as a result. The average period of follow-up was 17 months, ranging from 12 to 36 months. At the most recent follow-up, Harris scores for the hip joints of patients ranged from 82 to 89, with an average score of 86. The X-rays revealed that all fractures surrounding the femoral prosthesis had healed and that there was no looseness in the femoral prosthesis. None of the PHILOS license plates had expired. All patients were able to perform full-load walking, and pain and claudication in affected limbs were significantly reduced. Finite element analysis and mechanical testing of the Synbone femoral model revealed that the fixation effect of the PHILOS group was superior to that of the Cable group; consequently, PHILOS plates can be used to effectively fix fractures around the proximal femoral prosthesis.

**Conclusion:** PHILOS plates are initially used in the treatment of Vancouver B1 PFF, which may be a good choice due to their simpler operation, lower medical costs, and satisfactory clinical efficacy.

## Introduction

In China’s society, the incidence of myeloid joint diseases and fractures has increased as the aging process has accelerated. Artificial joint replacement is an effective treatment (Bhattacharyya et al., 2007; Bulatović et al., 2017; Gitajn et al., 2017), and periprosthetic femoral fracture (PFF) is one of the most severe complications following hip replacement (Caruso et al., 2018). Consequently, the Vancouver B1 fracture is the most prevalent type of PFF (Sah et al., 2010; Wähnert et al., 2017), accounting for approximately 75% of all PFF cases (OʼConnell et al., 2018), with a nonunion rate of 42% after conservative treatment (Moloney et al., 2016; Stoffel et al., 2020). The subsequent treatment of the aforementioned complications is difficult and ineffective. Increasing evidence demonstrates that early surgery, early rehabilitation, and early weight-bearing exercise can effectively reduce patient mortality (Caruso et al., 2018; Agostini et al., 2022). Currently, it is believed that surgery is the best treatment for the aforementioned complications, but there is no clear consensus regarding the optimal treatment strategy for them.

Common surgical treatments for Vancouver B1 fractures include locking plate internal fixation, cable (steel wire) internal fixation, and locking plate and cable internal fixation (Graham et al., 2015; Khan et al., 2017; Min et al., 2018). Otherwise, some investigators have used hook plates (Lenz et al., 2016) and distal femoral locking plates (Takahashi et al., 2021) to fixate Vancouver B1 fractures, but the results have been unsatisfactory. Since September 2017, the Department of Orthopaedics at Wujin Hospital, affiliated with Jiangsu University, has creatively applied PHILOS plates to the treatment of Vancouver B1 PFF, achieving excellent clinical efficacy and social benefits.

The mechanical stability of PHILOS plates in Vancouver B1 fractures is unknown. We used the three-dimensional finite element method and the mechanical test of the Synbone femoral model, the mechanical stability of PHILOS and the Cable Ready GTR titanium cable hook plate system was compared in the treatment of Vancouver B1 PFF, thereby providing a mechanical theoretical basis for the use of PHILOS in the treatment of Vancouver B1 PFF.

## Data and methods

### Clinical data

**FIGURE 1:**
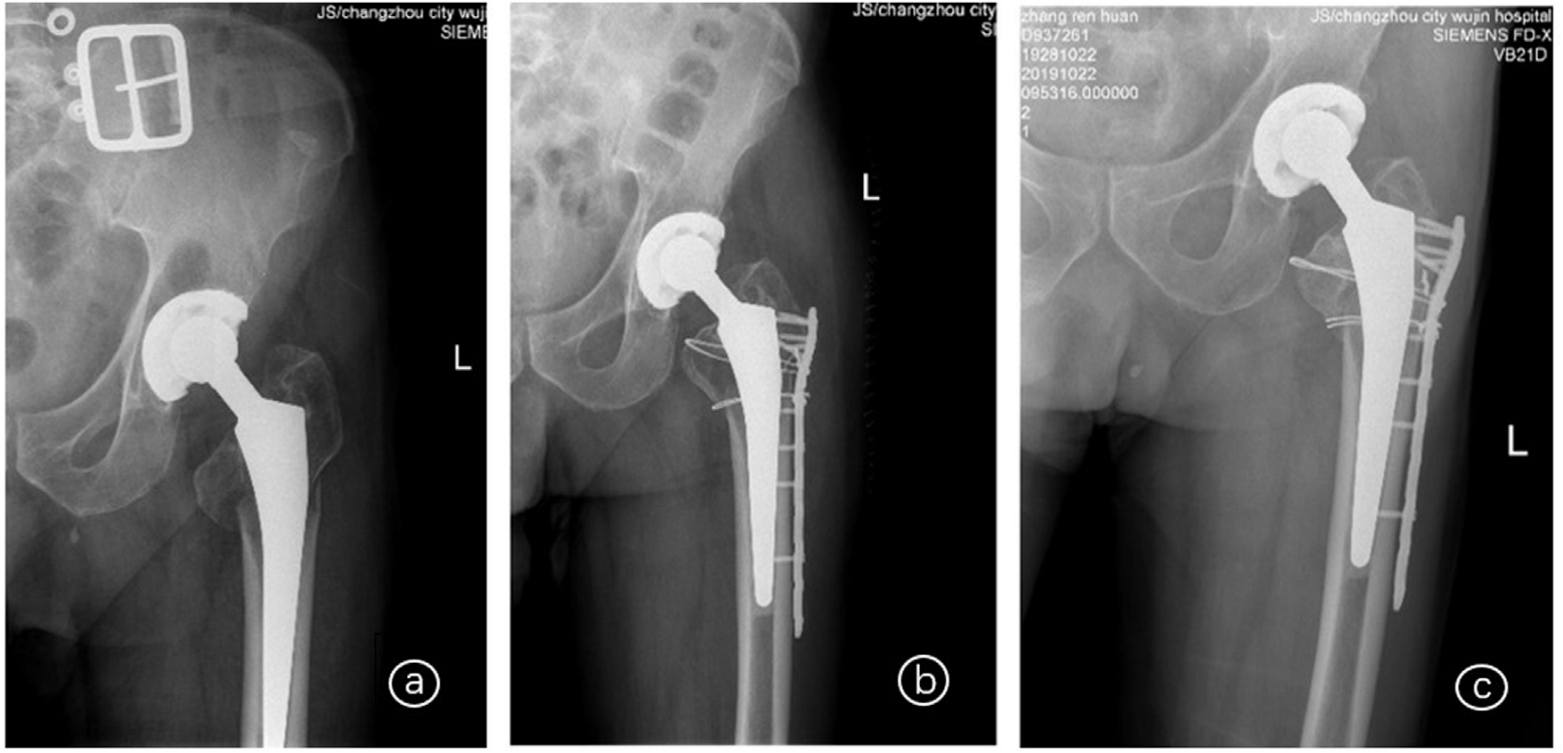
X-rays of an 86-year-old female patient with Vancouver B1 PFF who underwent left hip arthroplasty 14 years and 2 months ago; **ⓐ** Before surgery; **ⓑ** 5 days after surgery; **ⓒ** 1 year and 9 months after surgery.

In this study, there were 18 cases (18 hips), including 10 males and 8 females. Their average age was 72.61 ± 8.19 years, with a range of 57–86 years old. There were 11 cases of left hip arthroplasty and 7 cases of right hip arthroplasty among the 18 cases, and all hips were Vancouver B1. All 18 cases had PFF following hip arthroplasty. The initial hip replacement was performed due to a femoral neck fracture, and the DePuy total biological hip system with a Corail stem was utilized. In terms of the causes of the injuries, three cases were the result of automobile accidents, while the remaining cases were the result of falls. From 5 months to 14 years and 2 months had passed since the last hip replacement surgery, with a mean of 62.34 ± 58.28 months. Before the injury, none of the patients exhibited obvious limitations in hip joint mobility and were capable of caring for themselves. 3 cases were simultaneously complicated by diabetes, hypertension, and renal insufficiency; 4 cases were simultaneously complicated by diabetes and hypertension; and 10 cases were simultaneously complicated by hypertension. The hospital’s ethics committee approved the study, and all patients signed informed consent forms. All of the surgical procedures on the patients were performed by the same surgeon and team. The operation was performed under general anesthesia, and the patients were in a healthy lateral position. The modified Harding approach from the initial incision was utilized to expose the fractured end around the PFF, and the hip joint was extracted to evaluate the stability of the femoral prosthesis. As with the Vancouver B1 PFF, the fracture end was exposed and repositioned, and periosteal peeling should be kept to a minimum. 1-2 double-strand M650 steel wires were used for binding and fixing, and a PHILOS plate (Chuangsheng, Changzhou, China) of the proper length was then placed (Figure 1).

### Data scanning and modeling

A 34-year-old male volunteer with a height of 173 cm and a weight of 65 kg was chosen to undergo continuous thin-layer spiral CT scanning from the hip joint to the middle tibia. The voltage was set at 120 kV, the current was 150 mA, and the scanning layer thickness was 0.625 mm. The femoral model was reconstructed using the threshold segmentation, regional growth, and 3D reconstruction functions of Mimics21.0 software (Belgium Materialise Company), while the cancellous bone model was created using the 3-matic software (Belgium Materialise Company). The solid model of the femur was created using the software Geomagic 12.0 (Raindrop, United States). The Medical Ethics Committee of Wujin Hospital, affiliated with Jiangsu University, has approved this study (No. 2021-SR-002). The participants have signed the informed consent form.

### Fixed system modeling

**FIGURE 2:**
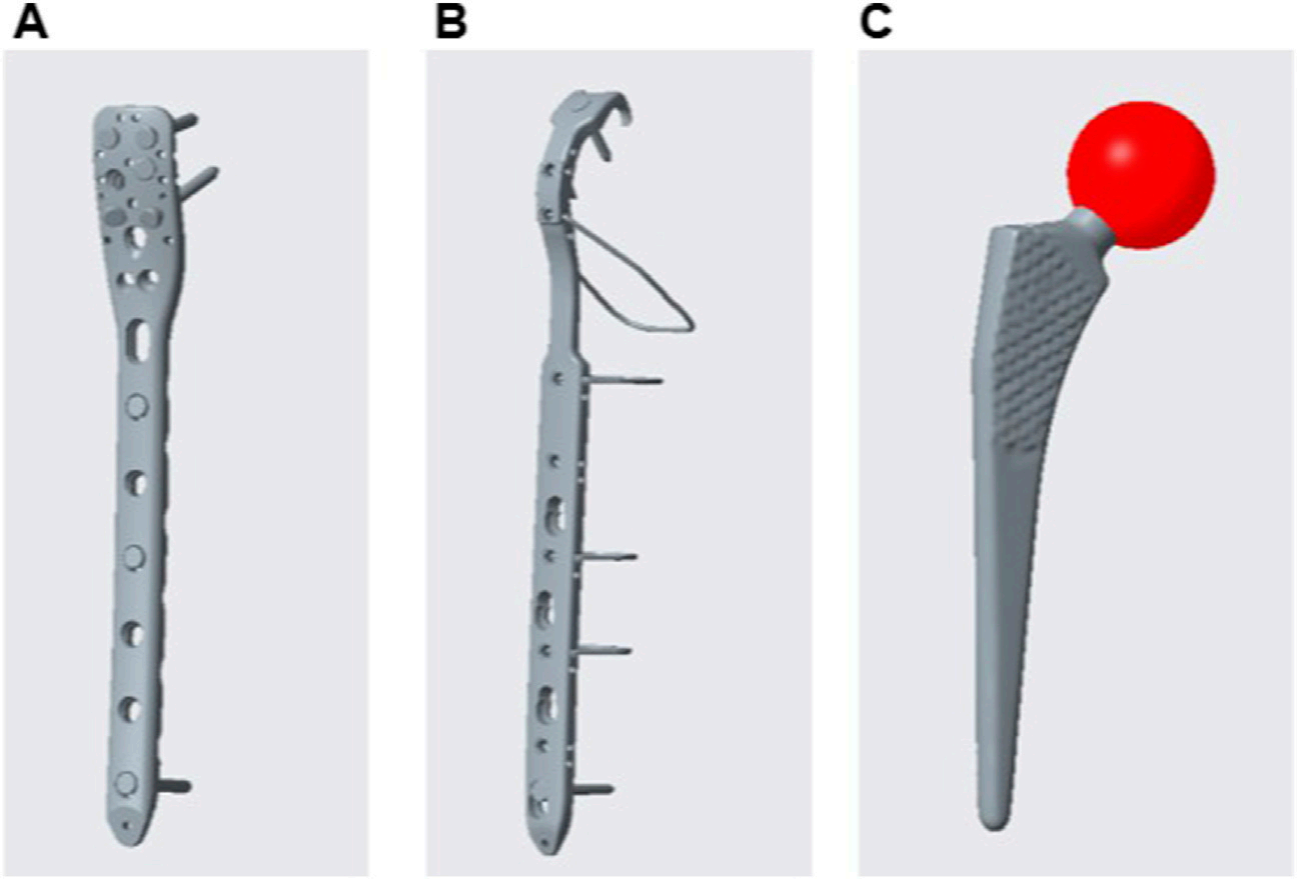
Internal fixation model: PHILOS **(A)**; Cable steel plate **(B)**; Femoral stem **(C)**; PHILOS is a proximal humeral locking plate.

The femur model was imported into Creo Parametric 5.0 (PTC, United States) software. In Creo software, the PHILOS and Cable Ready GTR titanium cable hook plate systems and the femoral stem prosthesis model were created, and the internal fixation was assembled with the femoral model to complete the grouping model of fixation after Vancouver B1 PFF (Figure 2).

### Processing of grid model

The assembled model was imported into the Hypermesh software, and a component was created for each model component. The Vancouver B1 PFF was simulated at the middle and upper positions of the femoral shaft, and the femoral shaft was then cut with a fracture line gap of approximately 0.2 mm to complete the finite element model of two fixation methods after Vancouver B1 PFF, namely, PHILOS fixation (PHILOS group) and Cable plate fixation (Cable group). Contacts were defined between all interfaces using contact element. Plate and bone were considered as “No separation condition” and all the other interfaces were set as “Bonded condition” (Raja et al., 2020).

**FIGURE 3:**
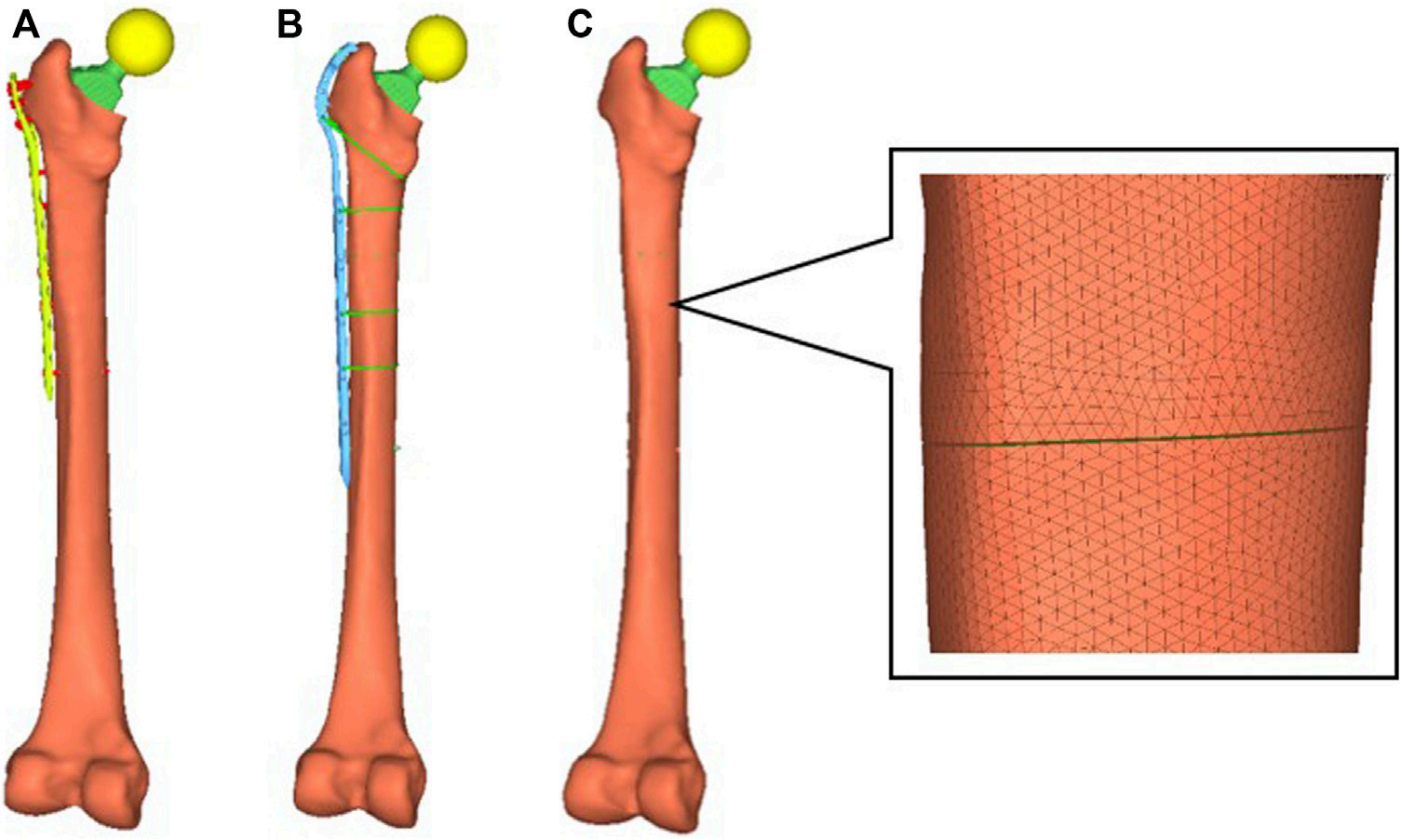
Model grouping. In PFF, the femoral shaft was situated at the end of the femoral stem. Two groups of models fractured at the same location, and PHILOS fixation and cable fixation, respectively, were performed. PHILOS group **(A)**; Cable group **(B)**; fracture position of the model **(C)**; Cable Ready GTR is a titanium cable hook plate system; PHILOS is a proximal humeral locking plate.

The femoral model is divided into the cortical and cancellous bone by the requirements of the literature. The volume mesh was divided using tetrahedron Solid 187 element mesh after the necessary editing and processing for each part. Figure 3 depicts that the PHILOS group had 1649969 nodes and 1034123 elements, whereas the Cable group had 1977961 nodes and 1236660 elements.

### Material property assignment

The corresponding material proper tie were assigned to each component of the model in Hypermesh software (Table 1), based on the literatures (Ma et al., 2014; Mei et al., 2014; Liang et al., 2017; Tianye et al., 2019; Cui et al., 2020).

**TABLE 1:** Elastic modulus and poisson’s ratio of each material.

Material	Elastic modulus (MPa)	Poisson’s ratio
Cortical bone	16,800.0	0.3
Cancellous bone	840.0	0.3
Femoral head	110,000.0	0.3
Femoral stem	110,000.0	0.3
Bone plate	110,000.0	0.3
Titanium cable	110,000.0	0.3

### Boundary conditions and loads

The femoral stress states are extremely complex. Under normal movement conditions, such as normal gait, the maximum load through the hip joint is approximately 2.6–4.1 times (Chang et al., 2015) an individual’s body weight. The load on the hip joint increases with an increase in step speed, step length, or body weight. Furthermore, the muscle force on the femur is extremely complex. Taylor et al. (1996). Believed that the muscle loading of the femur model was subject to numerous uncertainties, including the selection of muscle quantities, gravity, and the direction of the muscle force loading. Particularly, it is nearly impossible to simulate the load accurately and completely under dynamic conditions. To simplify the analysis and highlight the fixation effect of the two groups of models, the femoral prosthesis head was used to simulate the cases of a human standing on two legs, standing on one leg, and ascending stairs, with vertical forces of 700, 1,400, and 2,100 N, respectively (Cui et al., 2020; Wang et al., 2020). All distal lymph nodes below the femoral condyle were fully constrained.

### Effectiveness validation

To verify the efficacy of the model, a complete femoral model was constructed initially. Material properties were assigned based on research (Papini et al., 2007). The model’s lower end is fully constrained, and a vertical load of 1,500 N was applied to the femoral head. The model was analyzed using Ansys19.0 software (ANSYS Company, United States), and the results were compared to those in the literature.

### Biomechanical evaluation of a synthetic bone model

**FIGURE 4:**
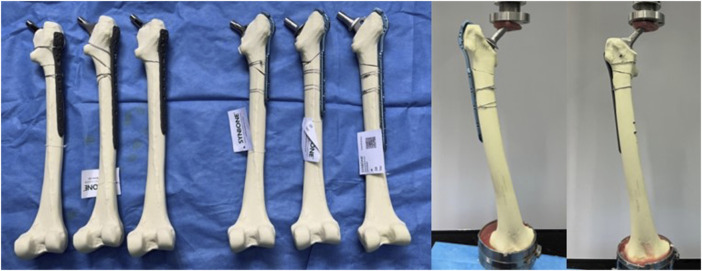
Synbone femoral model with PHILOS and Cable GTR plates implanted for fixation, respectively.

Six Type 2,200 femur models from Synbone were purchased. The femoral trochanter was modeled with a Vancouver B1 fracture and then divided into two groups that were fixed with PHILOS and Cable hook plates, respectively (Figure 4). According to the cited sources, a new set of femoral test fixtures was created. The distal end of the femur was placed in a custom fixture to simulate the contact force vector during the single-leg standing phase, and the lower end was embedded in dental support powder with an abducted femur and an 8° force line. The upper end of the femoral stem was embedded with bone cement, preventing the femur from moving in all directions during the loading process. Utilizing an Instron E10000 tensile torsional biaxial universal material mechanics testing machine, the test was conducted. The software for static testing was Bluehill 2.0, and the software for dynamic testing was WaveMatrix.

Adjustments were made to the distance between the upper and lower indenters so that the femur was perfectly vertical, with an 11° angle between the femoral shaft and the force line. The cycle test was conducted initially with 0–500 N, 2 Hz, and 2,000 cycles (Pletka et al., 2011; Kemker et al., 2017). After the fatigue test, the load-displacement curve was recorded, and the dislocation displacement of the fracture line was measured. The displacement changes of the femur before and after the test cycle were analyzed statistically. The primary objective of this step was to eliminate the space between the femur and the fixture, as well as the displacement of the femoral neck fracture slip. After the cycle was completed, compression loads of 700 N, 1,400 N, and 2,100 N were applied at a rate of 3 mm/min until the model was damaged or the compression load reached 2,100 N (approximately three times the weight of a 70 kg adult). The load-displacement curve was recorded, and the fixation effect of each group’s models was compared by calculating stiffness.

### Evaluation indexes

After surgery, patients were followed up on as outpatients in the first, third, sixth, and 12th months, and then every 6 months. At each follow-up, X-rays were taken to determine if the fracture had healed, if the internal fixation had failed, and if the femoral prosthesis was loose. Each follow-up’s Harris score was recorded. The total Harris score is 100 points, with 44 points assigned to pain, 47 to function, 4 to deformity, and 5 to mobility. ≥ 90 indicates Excellent, 80–89 indicates Good, 70–79 indicates Acceptable, and <70 indicates Poor.

Ansys 19.0 simulation software was used to observe the distribution of the Von Mises maximum stress and the maximum deformation in models of the two groups subjected to 700 N, 1,400 N, and 2,100 N load conditions. Perioperative data were recorded. The Harris score of the hip joint was used to evaluate clinical outcomes and imaging tests were performed routinely. It was determined if the fracture had healed, if the internal fixation had failed, and if the femoral prosthesis was loose.

### Statistical analysis

The SPSS 13.0 program was utilized for statistical data analysis. The measurement information was represented as ( $\overset{®}{x}\pm s$ ). One-way ANOVA was used to examine Harris scores at various follow-up periods. *p* < 0.05 indicates a statistically significant difference.

## Results

### Clinical results

In this study, 18 patients successfully underwent surgery; the duration of the operation ranged from 65 to 130 min, with an average of 105.00 ± 25.52 min; and intraoperative bleeding ranged from 150 to 650 mL, with an average of 303.39 ± 120.80 mL. There was no intraoperative blood transfusion. After surgery, there were no serious complications such as infection or deep vein thrombosis. All patients were monitored for a period of 12–36 months, with a mean of 17.29 ± 3.86 months. The difference is statistically significant (P0.001) from 1 month after surgery (54.33 ± 5.50 points) to 3 months after surgery (67.67 ± 5.32 points) to 6 months after surgery (78.67 ± 3.33 points) to 12 months after surgery (84.67 ± 3.08 points) to the last follow-up (86.00 ± 2.28 points); the difference is statistically significant (*p* < 0.001). At the most recent follow-up, 15 patients were able to walk without assistance, while 3 patients required a walker for full-load walking. In terms of imaging examination, successive callus was observed at the end of the PFF 3 months after surgery, and the PFF attained the bone healing standard 6 months after surgery without invalidation of the PHILOS plate and looseness of the femoral prosthesis.

### Results of validation

**FIGURE 5:**
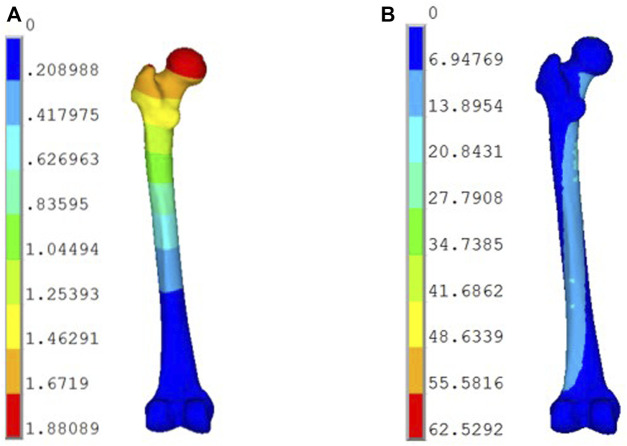
Displacement and stress distribution of the femur under physiological conditions: **(A)** Displacement Distribution (mm); **(B)** Stress Distribution (MPa).

The compression stiffness of the complete model under 1,500 N of pure compression was 0.798 kN/mm (Figure 5), which was very close to the compression stiffness [(0.76 ± 0.26) kN/mm] of the mechanical test reported in the literature (Papini et al., 2007). Taking into account the unique characteristics of each model, the models developed in this experiment were effective.

### Maximum deformation of the model

**FIGURE 6:**
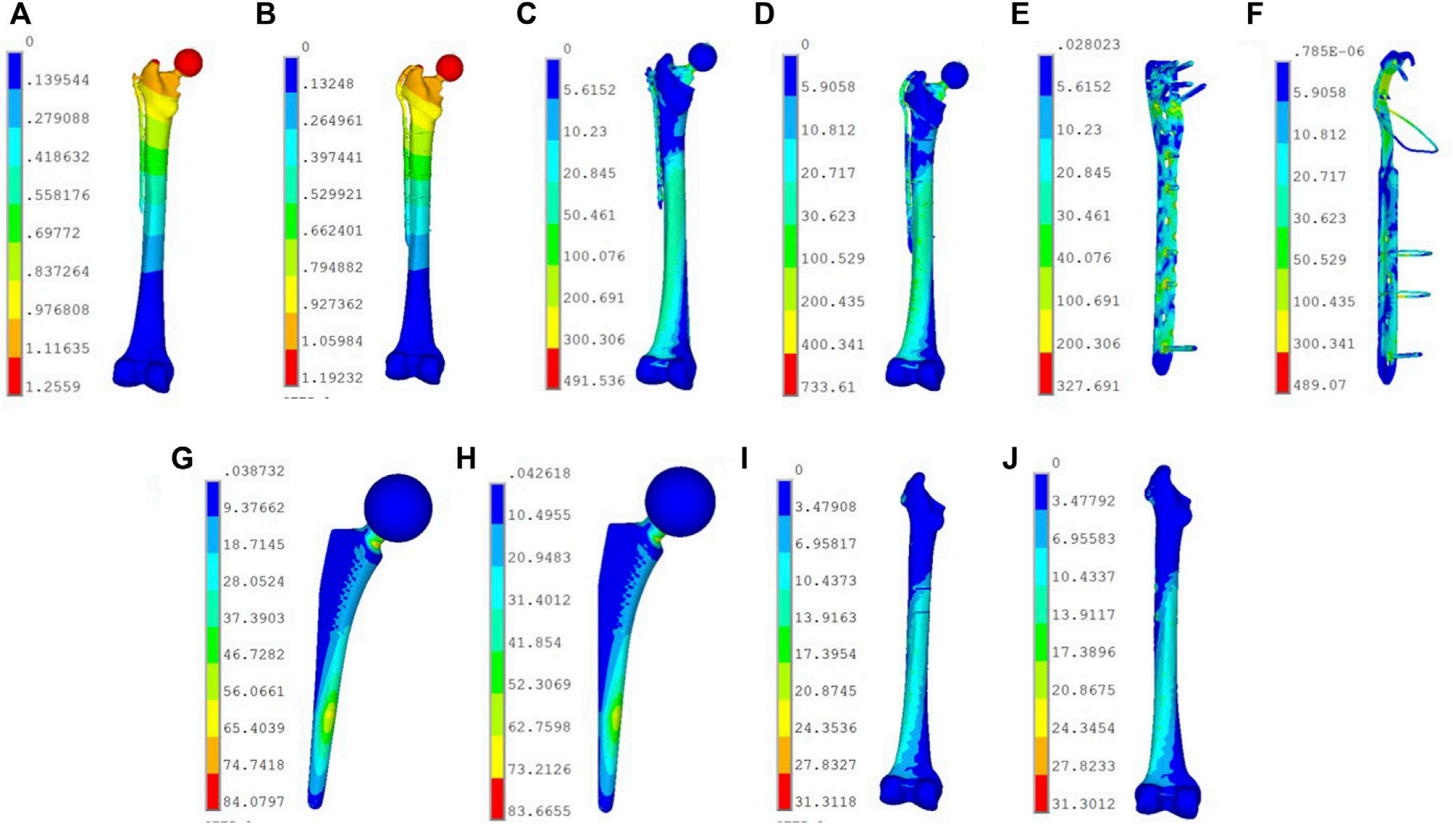
Displacement and stress distribution of PHILOS group and Cable group models. **(A)** Deformation distribution of PHILOS group (mm). **(B)** Deformation distribution of Cable group (mm). The maximum deformation of this two models in the Cable group under 700 N load. **(C)** Stress distribution of PHILOS group (MPa). **(D)** Stress distribution of Cable group (MPa). Stress distribution distribution of the overall model in the two groups under 2,100 N load. **(E)** Stress distribution of the plate in PHILOS group (MPa). Stress distribution distribution of fixed steel plate in the PHILOS group under 1,400 N load, with the maximum stress peak value of 327.69 Mpa, which is distributed on the fixed screw. **(F)** Stress distribution of the plate in Cable group (MPa). Stress distribution distribution of fixed steel plate in Cable group under 1,400 N load, with the maximum stress peak value of 489.07 Mpa, which is distributed on the second titanium wire under the fracture. **(G)** Stress distribution of femoral stem in PHILOS group (MPa). Stress distribution distribution of the femoral stem in the PHILOS group under 700 N load, with the maximum stress peak value of 83.45 Mpa, which is distributed at the neck of the femoral shaft. **(H)** Stress distribution of femoral stem in Cable group (MPa). Stress distribution distribution of the femoral stem in the Cable group under 700 N load, with the maximum stress peak value of 84.08 MPa, which is distributed at the neck of the femoral shaft. **(I)** Stress distribution of the bone model in PHILOS group (MPa). Stress distribution distribution of the femoral shaft in the PHILOS group under 700 N load, with the maximum stress peak value of 31.31 Mpa, which is distributed on the inner side of the femoral shaft below the fracture line. **(J)** Stress distribution of the bone model in Cable group (MPa). Stress distribution distribution of the femoral shaft in the Cable group under 700 N load, with the maximum stress peak value of 31.31 Mpa, which is distributed on the inner side of the femoral shaft below the fracture line.

Consistent with the location of load application, the maximum deformation of the models in both groups occurred on the prosthesis ball head. Since the material properties assigned to the femur, prosthesis, and internal fixation system were isotropic, the results demonstrated a linear relationship between the load and the deformation of the models in the two groups. The maximum deformation of the PHILOS group was 3.77 mm when the compression load was 2,100 N, which is greater than that of the Cable group (3.58 mm) (Figures 6A, B). Under a 1,400 N load, the PHILOS group and the Cable group deformed by 2.51 mm and 2.38 mm, respectively. When the load was 700 N, the difference in deformation between the models in the two groups was the greatest, and the maximum deformation in the PHILOS group was 5.8% greater than that in the Cable group (1.19 mm). However, there was no difference in the distribution of deformations on the fixed steel plate between the two groups of 1.05 mm models. It can be concluded that the two model groups possess comparable mechanical stability.

### Stress distribution of the models

With an increase in compression load, the model’s maximum stress also increased. Under a 2,100 N load, the PHILOS group’s maximum peak stress was 491.54 MPa, while the Cable group’s was 733.61 MPa (Figures 6C–J). Under three types of loads, the PHILOS group experienced approximately 49.2% less stress than the Cable group. Consequently, the maximum stress peak in the PHILOS group was predominantly distributed at the fourth and fifth fixed ends of the plate, whereas the maximum stress peak in the Cable group was predominantly distributed on the second titanium cable below the fracture line, while the stress on the other titanium cables was lower. According to the analysis of the stress nephogram distribution or stress peak, the stress distribution on the cortical bone, cancellous bone, and femoral shaft of the two groups of models was extremely similar. Nonetheless, the stress distribution on the prosthesis in the Cable group was slightly higher than that in the PHILOS group (Table 2), indicating that the analysis model was primarily scientific.

**TABLE 2:** Stress value (MPa) of the femur and femoral stem in models of PHILOS group and cable group under 700 N, 1400 N, and 2100 N loads.

Load (N) stress on the femur			Stress on the femoral stem	
	PHILOS group	Cable group	PHILOS group	Cable group
700	31.30	31.31	83.67	84.08
1,400	62.62	62.62	166.91	168.16
2,100	93.90	93.94	250.36	252.24

### Biomechanical test of the artificial bone model

After the fatigue test with 0–500 N was completed, the results from the scale plates on both sides of the fracture line indicated that the fracture line displacements in the two groups of specimens were as follows: 1.06 ± 0.47 mm for the PHILOS group and 1.63 ± 0.90 mm for the Cable group (*p* = 0.615). As determined by the displacement sensors on the indenters, the overall deformations of the specimens were as follows: 1.77 ± 0.55 mm for PHILOS and 1.49 ± 0.53 mm for Cable (*p* = 0.253).

Following the fatigue test, static compression tests were conducted to apply compression loads of 700 N, 1,400 N, and 2,200 N to the specimens, thereby obtaining the static compression deformation. The compression rigidity was computed. Each group of specimens was examined three times, and the average values were then determined. Following are the values for compression stiffness under 700 N compression: 294.50 ± 89.07 N/mm for the PHILOS group and 275.16 ± 74.87 N/mm for the Cable group (*p* = 0.716); 233.14 ± 67.08 N/mm for the PHILOS group and 299.97 ± 25.69 N/mm for the Cable group (*p* = 0.200); 195.76 ± 34.49 N/mm for the PHILOS group and 180.51 ± 65.12 N/mm for the Cable group (*p* = 0.070).

## Discussion

Since 1993, the incidence of periprosthetic fractures after hip arthroplasty has increased from 0.1% to 2.1% (Bulatović et al., 2017). The majority of these fractures occur in elderly patients, who frequently suffer from a variety of chronic diseases and osteoporosis. The Vancouver classification of PFF has treatment implications (Caruso et al., 2018; Agostini et al., 2022). Incision, reduction, and internal fixation are the treatment tenets for type B fractures. However, due to the complexity of the operation and the occurrence of numerous complications, there is no gold standard for the selection of internal fixation, and the surgical effect is debatable. Internal fixation options currently include primarily dynamic compression plate, memory alloy embracing device, steel cable encircling, and locking plate. Tsiridis et al. (2005) utilized a DCP plate to treat 18 cases of Vancouver B PFF, 11 of which were healed at the final follow-up after an average of 13 months. Nonunion was observed in 4 cases, and 3 patients died without observed fracture healing. Bulatović et al. (2017) found that the average hospital stay for patients with Vancouver B PFF was lengthy, and the modified Merle d’Aubigne score was low after surgery. Graham et al. (2015) reported that in biomechanics, the combination of locking nails and steel cable was more advantageous.

Chatziagorou et al. (2019) believed that the reoperation rate of Vancouver B PFF patients treated with conventional plating or locking plating after surgery was identical. Sah et al. (2010) reported that the efficacy of a single locking plate in the treatment of Vancouver B PFF was satisfactory, but they emphasized the importance of soft tissue cuff protection and adequate cortical bone screw fixation. Min et al. (2018) suggested a minimally invasive method for implanting a locking plate to treat Vancouver B1 PFF. This technique resulted in fewer intraoperative complications, and its postoperative imaging examination and clinical efficacy was comparable to those of incision reduction and internal fixation.

Due to the presence of the femoral stem prosthesis in the proximal femoral medullary cavity, implanting crews, particularly bicortical screws, is difficult. These patients are frequently afflicted with osteoporosis, which makes the control of screws extremely difficult. Lunebourg et al. (2015) created a non-locking arc plate with eccentric holes to avoid intramedullary femoral stem prosthesis and achieve bicortical screw fixation, thereby enhancing the fracture end’s stability. However, the placement position of the plate was challenging, and the fracture end fixation was not reliable, necessitating customization. Wahnert et al. (2017) demonstrated that the application of double plates in the Vancouver B1 PFF could significantly enhance the structural strength of internal fixation and fracture end stability. Similar clinical research results were reported by O'Connell et al. (2018), but the internal fixation with a double plate has the disadvantages of larger surgical trauma, more bleeding, greater irritation of local soft tissue, and higher medical costs. The biomechanical study by OʼConnell et al. (2018) confirmed that Vancouver B1 PFF with osteoporosis was fixed with bicortical screws at the proximal end of the femur, permitting early weight-bearing even without the reinforcement of an allograft bone plate. Stoffel et al. (2020) and Moloney et al. (2016) found that the clinical efficacy of a locking plate combined with an allograft bone plate in the treatment of Vancouver B1 PFF was adequate, but there was a high risk of infection and infectious disease transmission.

The anatomical outline of the lateral side of the proximal femur was quite similar to that of the lateral side of the proximal humerus, as determined by careful examination of the proximal femur’s structure. Therefore, we applied the PHILOS plate creatively to Vancouver B1 and BFF PFF. At the proximal end of the PHILOS plate, there are A, B, C, D, and E holes, with a total of 8 locking holes, 1 LCP bonding hole, and 10 suture holes. When a femoral stem prosthesis occupies the proximal femoral medullary cavity, the implantation of sufficient and long locking nails is still possible, and even some locking nails are permitted to be implanted in the bicortical cortex, due to the presence of nine locking holes in different directions at the proximal end of PHILOS. Therefore, even in osteoporosis patients, it can still offer a high pull-out resistance. The presence of the suture hole permits suturing and fixing the fracture piece with blood supply around the fracture end, or transplanting iliac allograft to promote fracture healing, as well as providing auxiliary steel wire perforation fixation to prevent steel wire slip failure and suturing the surrounding muscles and tendons to neutralize muscle force. Multiple long and short locks at the proximal end of the PHILOS plate were staggered on seven patients in this study to fix the fracture end around the femoral prosthesis. Because the fracture end was reliably fixed and the blood supply to the fracture end was safeguarded as much as possible, patients were permitted to descend to the ground shortly after surgery, without serious complications or fracture nonunion. In addition, the Harris scores for postoperative follow-up indicated that the application of the PHILOS plate in Vancouver B1 PFF contributed to a speedy recovery. The patient’s hip joint function stabilized 12 months after surgery and could be further enhanced with time extension. PHILOS is a single-locking steel plate with a small implant volume and minimal soft tissue irritation; therefore, double plate fixation, allograft bone plate strengthening fixation, and special steel cable encirclement were not required. Thus, it has clear benefits for controlling medical costs.

The fracture around the proximal femoral prosthesis was simulated using the finite element model of the femur obtained from the two-dimensional continuous CT data of volunteers and medical engineering software such as Mimics, Geomagic, Hypermesh, etc. There were two distinct surgical fixations utilized. Three-dimensional finite element models of two groups of internal fixation were given the same load and constraint conditions to simulate the single-leg standing condition of adult men. In conclusion, the biomechanical properties of various fixation techniques were compared and analyzed. Using the Ansys19.0 software, simulation calculations were performed to observe the maximum stress distribution and maximum deformation of the models in two groups under various load conditions.

Under various loads, the deformation value of the femur-cab group was less than that of the femur-philos group, which indicated that the mechanical stability provided by the femur-cab group was slightly better than that of the femur-philos group. Under various loads, the stress values on the femoral stem and femur of the two groups of models were comparable, indicating that both groups could provide adequate mechanical stability. Regarding the model’s stress distribution, the stress of the femur-cab group was significantly higher than that of the femur-philos group, with the majority of the stress being distributed on the second titanium alloy binding wire. The overall conclusion is that the fixation effect of the femur-philos group is superior to that of the femur-cab group, making it a more effective method for PFF. In the range of 0–700 N load, the Philos group demonstrated superior mechanical properties compared to the Cable group; in the range of 700–1,400 N load, the Cable group demonstrated superior mechanical properties compared to the Philos group; and in the range of 1,400–2,100 N load, the Philos group demonstrated superior mechanical properties compared to the Cable group. Thus, under normal body exercise loads, the PHILOS plate’s fixation effect is more reliable.

There are still some inadequacies of this study. As the biomechanical test conditions are limited, we cannot take into account the muscle forces around the femur (Amirouche et al., 2016) in the loading process during the biomechanical test and finite element analysis. Bone remodeling is an important clinical parameter in fracture healing and also is one of the major concerns of surgeons. Unfortunately, we have not studied the relationship between fracture healing and the different number of screws on the plate and the position of the screws. Simultaneous simplified methods of screw placement and threading were used for plate fixation, without the consideration of torque and extrusion forces that increase fracture fixation strength (Bronsnick et al., 2015; Mejia et al., 2018; Addevico et al., 2020; Addevico et al., 2021). In recent years, with the development of mechanical and biomechanical techniques (Travascio et al., 2021), the use of coated screws has increased the osteogenic effect, particularly in patients with osteoporosis (Patel et al., 2015), while increasing the screw-to-bone friction, which is critical for implant fixation and implant stability (Bronsnick et al., 2015).

In conclusion, our preliminary study demonstrates that the PHILOS plate can provide stable fixation for the treatment of Vancouver B1 PFF, with the benefits of good fracture healing, simpler operation, lower medical costs, and satisfactory clinical effect. Due to the small number of cases in this study and the brief follow-up durations, the definitive treatment effect must be confirmed on a larger scale.

## Data Availability

The original contributions presented in the study are included in the article/Supplementary material, further inquiries can be directed to the corresponding author.
